# Sauna Yoga Superiorly Improves Flexibility, Strength, and Balance: A Two-Armed Randomized Controlled Trial in Healthy Older Adults

**DOI:** 10.3390/ijerph16193721

**Published:** 2019-10-02

**Authors:** Heidi Bucht, Lars Donath

**Affiliations:** 1 Institute of Sport and Movement Gerontology, German Sport University Cologne, 50933 Cologne, Germany; heidibucht@gmail.com; 2 Institute of Exercise Science and Sport Informatics, German Sport University Cologne, 50933 Cologne, Germany

**Keywords:** thermal therapy, elderly, body–mind, stretching, resistance training, postural control, quality of life

## Abstract

Besides strength and balance, flexibility is an important indicator of health-related physical fitness. Thus, the aim of this two-armed randomized controlled pilot trial was to investigate whether sauna yoga at a moderate temperature (50 °C) beneficially affects flexibility, strength, balance, and quality of life (QOL) in healthy elderly community dwellers. Participants were randomly assigned to an intervention group (INT, *n* = 11, age: 68.7 ± 5.9) or control group (CON, *n* = 12, age: 69.3 ± 4.9), using the minimization method. Age, physical activity, gender, and the primary outcome flexibility were used as strata for group allocation. Both groups completed similar exercises in the sauna over eight weeks. Only the INT group was exposed to moderate temperatures of 50 °C. Large and statistically significant improvement in favor of the sauna group (INT) was observed for the chair sit-and-reach test (INT: +83%, CON +3%, *p* = 0.028, n_p_^2^ = 0.24). The shoulder and lateral spine flexibility were not relevantly affected. Strength in the lower extremities merely showed a tendency to significant changes (INT: 16%, CON: 3%, *p* = 0.061, n_p_^2^ = 0.181). Additionally, balance abilities, with eyes closed, improved (INT: 187%, CON +58%, *p* = 0.056, n_p_^2^ = 0.189) in favor of the INT group. QOL only improved in favor of the INT for environmental dimension (INT: +7%, CON: 0%, *p* = 0.034, n_p_^2^ = 0.227). These first but preliminary findings indicate that sauna yoga may serve as a promising and feasible means to improve flexibility in elderly people. Strength and balance do not meaningfully benefit from a sauna environment, although strength improved to a slightly higher extent in the sauna group. Future large-scale research is needed to elucidate underlying mechanisms and corroborate these findings.

## 1. Introduction

Along with strength, balance, and endurance, flexibility is considered an important physical dimension for active and healthy aging. In this regard, Nelson and coworkers reported that “to maintain the flexibility necessary for regular physical activity and daily life, older adults should perform activities that maintain or increase flexibility on at least two days each week for at least 10 min each” [1]. Yoga was shown to beneficially affect physical function and well-being in seniors and can thus be considered an appealing and easily applicable activity for this population [2]. Sauna, on the other hand, delivers a warm and relaxing environment, where the body and mind can be trained simultaneously. From a risk–benefit stand point, and in the light of stress, sleep, and mental benefits, only very few adverse events were reported [3]. Culturally, the sauna is very popular in Scandinavia, particularly in Finland, but it also strongly belongs to a worldwide spa culture. Many fitness and sports clubs provide saunas that are easily assessible.

Even though the potentially beneficial effects of both sauna and yoga are independently well studied and reported, there is hardly any literature concerning training on health-related surrogates of physical fitness, such as flexibility when employing a sauna with moderate temperatures. Training in a warm environment, however, was reported to improve the range of motion (ROM) [4,5], accompanied with reductions in perceived pain [6]. One acute study led by Leung and colleagues [7] interestingly reported acute responses after Qigong training in a Sauna: Leung et al. [7] observed higher heart rates of 30–40% above the pre-exercise level, whilst blood pressure, on the contrary, remained stable or even slightly decreased after a single Qigong session. 

Thus, sauna yoga might serve as a comprehensive body–mind exercise approach which aims to improve spine and shoulder flexibility, accompanied with exercises that strengthen the muscles in the trunk and lower extremities by using slow-paced stretching and strengthening exercises in a quiet and mildly warm environment. One sauna yoga session lasts 30 min and is performed in a sauna room with a temperature of approximately 50 °C. To the best of our knowledge, however, no longitudinal intervention study investigating the effects of combining the sauna with yoga on health-related physical fitness parameters was published. 

Against this background, the present two-armed randomized controlled trial investigated the effects of sauna yoga on flexibility as a primary outcome and strength, balance, and quality of life as secondary outcomes. We hypothesized that one weekly sauna yoga session over eight weeks would lead to superior improvements in flexibility compared to yoga performed in an ambient room temperature. As a secondary hypothesis, we assumed that strength, balance, and quality of life does not superiorly improve after sauna yoga. 

## 2. Materials and Methods

### 2.1. Study Design and Participants

**Figure 1:**
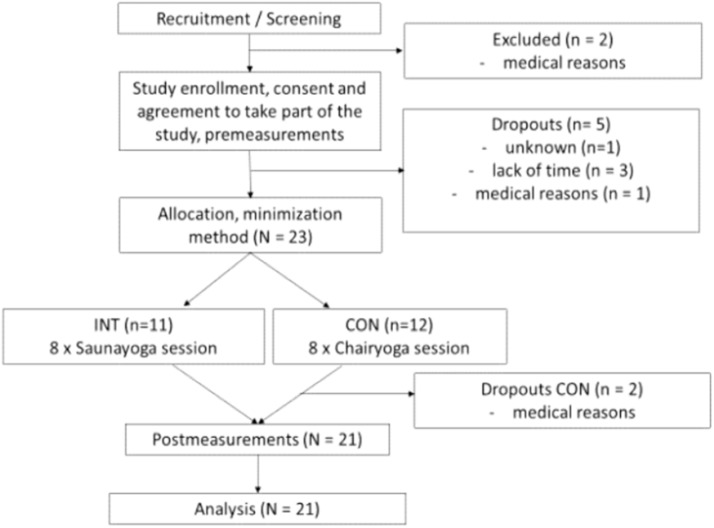
Participant flow throughout the study.

The study was designed as a two-armed randomized controlled study with pilot character (Figure 1). One weekly sauna session over 8 weeks was delivered. Twenty-three healthy and active older adults ranging from 60 to 80 years of age participated in this intervention study (Table 1). Recruitment for the study started in April 2019 by distributing flyers in fitness and sports clubs, senior cafés, and churches. All participants read and signed an informed consent document prior to participation. Before starting the intervention, a medical certificate on current mental and physical health status was requested from all participants. Participants with diagnosed acute or chronic disease, heart disease or pacemaker, medical surgery in the past 3 months, and with neurological or psychological disorders were excluded. The study was approved by the local ethical committee of the German Sport University Cologne (Approval number 062/2019) and complied with the ethical principles of the Declaration of Helsinki. The participants were either allocated to the intervention group (INT) or control group (CON) by using the minimization method [8] after pre-testing. Age, sex, physical activity (PAQ 50+) and flexibility (CSR) of the participants served as strata.

### 2.2. Intervention

The intervention was conducted during May and June 2019 and was followed by post-testing and data analysis in July. One sauna yoga session lasted 30 min and was conducted in a moderate temperature of 50.5 ± 2.4 °C across all sessions. The session consisted of six yoga poses (modified eagle pose, modified sun salutation, modified warrior pose, spine rotations, core 1 and 2, and modified lotus pose), and the majority of them were performed in a seated position. The training session started and ended with one minute of relaxation exercises for preparing the body and mind for the workout and calming down afterwards. In the meditation–relaxation part, the participants were requested to close their eyes and take a comfortable sitting position. The main sauna yoga part included four stretching and mobilizing movements: Shoulder and hip flexibility, spine rotation, flexion, and lateral flexion. Each stretching exercise was performed once, twice, or four times on each side, depending on the exercises (Table 2). In addition, two strengthening movements, focused on the muscles of the trunk and lower limbs, were also performed once to four times on each side. Static poses were held for 4 or 5 breaths, ending up approximately with 30 s each. Detailed information on duration, repetitions, and sets are depicted in Table 2. The exercises and their order were always kept similar during the entire intervention, and a slight progression was included by lengthening the time stayed in stretching poses, by increasing the number of sets, or by verbal encouragement to stay deeper and longer in static strengthening poses. The Instructor always showed the basic poses or exercises initially and then provided variation in order to increase exercise intensity. The control group underwent exactly the same training protocol, with similar exercises, times, durations, and repetitions in an ambient, non-heated sauna environment.

### 2.3. Testing Procedure

Pre-testing was conducted one week before the beginning of the intervention, and post-testing was conducted in the week after intervention cessation. The assessor, weekday, and time of pre- and post-measurements were kept similar. However, the test administrator was not blinded. The participants were given instructions before the measurements to guarantee a standardized measurement of all participants, and 2–3 familiarization trials were allowed. A standardized protocol was used. The tests used are described below.

### 2.4. Anthropometric Data

Prior to the start of the intervention, the body weight (kg) and height (cm) of the participants were measured. Subsequently, the body mass index (BMI) was calculated. Age (yr), gender, and weekly physical activity (German PAQ50+ -questionnaire) were also recorded.

### 2.5. Weekly Physical Activity

At the beginning of the data collection, the participants filled out the German PAQ-50+ -questionnaire [9]. This questionnaire is designed to assess physical activity level. The questionnaire refers to the duration and intensity of the average weekly physical activity and is specially modified for older groups. The test–retest reliability of total physical activity time (r = 0.60) and total energy expenditure (r = 0.52) can be considered acceptable [9]. The hours of physical activity are transformed to MET (metabolic equivalent of the respective task) scores, which express the energy expenditure related to body weight in physical activity as the multitude of the resting metabolic rate. The reported MET values were used as strata during minimization of the participants to INT and CON, respectively.

### 2.6. Posterior Muscle Chain Flexibility

The chair sit-and-reach (CSR) test of the Senior Fitness Test Manual by Rikli and Jones [10] was employed in order to measure flexibility of the posterior muscle chain, more specifically in the lower back and hamstrings. Therefore, the participants were requested to sit on a chair, which was placed against the wall for safety reasons. One leg was extended forward so that the heel remained on the floor. The ankle was bent 90°. The other leg needed to be flexed in a way that the foot could be placed flatly on the floor. The test administrator instructed the subject to bend toward the toes, keeping the back and the extended leg straight. At the endpoint, the subject had to remain stretched for 2 s. The distance between fingertips and toes was measured. The score was noted as negative when the fingers and toes did not touch and positive if they did overlap. The test was performed twice. The best trial in centimeters to one decimal point was noted. CSR testing provides good validity and intraclass test–retest reliability (ICC, r = 0.92 for men; ICC; r = 0.96 for women), and it better correlates to hamstring flexibility in elderly people than the floor sit-and-reach test does [11]. Further studies indicate that the CSR test produces reasonably accurate and stable measures of hamstring flexibility [12].

### 2.7. Shoulder Flexibility

The flexibility of shoulders was measured using the back scratch (BS) test of the Senior Fitness Test Manual by Rikli and Jones [10]. The test was conducted in standing position. The subject placed one hand behind the back and the other over the shoulder, behind the head. Participants were asked to reach as far as possible, attempting to overlap the middle fingers of both hands. The test administrator measured the distance between the fingers. The score was positive when the hands overlapped each other and negative when the hands stayed apart from each other. The test was performed twice, and the best trial was noted to one decimal point. The BS is reported to have a good intraclass test–retest reliability (ICC, r = 0.98) [13] and is regarded as a valid instrument for measuring the upper-body flexibility of older adults [14].

### 2.8. Lateral Flexibility of the Spine

Mobility of the spine was measured by using the lateral flexion (LF) test. The subject stood with the back against the wall and feet 20 cm apart from each other. The subject was instructed to bend laterally as far as possible without rotating the upper body. The palm of the hand needed to be placed laterally on the thigh. In the maximal bending position, the distance between the floor and the middle finger was captured. The percentage change of pre- and post-measurements were included into the subsequent statistical analysis. The test was conducted twice on each side. The best scores on either side were noted in centimeters to one decimal point. Spinal mobility is usually assessed by using easy techniques [15]. LF is a valid test to measure the spine’s flexibility, and it has good interclass and intraclass test–retest reliability (ICC, r = 0.98) [16]. Frost, Stuckey, Smalley, and Dorman [17] identified the special effects of stretching and learned that the LF test can be used to qualify progress or control the effects of the intervention.

### 2.9. Strength in Lower Extremities

The five times sit-to-stand test (5STS) of the Short Physical Performance Battery (SPPB) [18] was used to measure participants’ strength in the lower extremities. It is considered a valid method [19], with excellent intra-rater (ICC, r = 0.97), as well as test–retest reliability (ICC, r = 0.99). The 5STS test associates significantly with the muscle strength of the lower extremities [20]. The participant sat in an armless chair with a seat height of 43 cm. The participants were instructed to hold their arms crossed over their chest and to sit by holding their back against the backrest of the chair, if possible. The soles of the feet were in contact with the ground, and the knees were bent to 90°. The subject then had to stand up from the chair to a full standing position and to extend hips and knees. The time started from the test administrators’ command “go” onward and was recorded using a stopwatch when the subject sat the fifth time on the chair and the back was against the backrest. Initial instruction was to perform the test “as quickly as possible”. The test was repeated twice, and the best time in seconds to one decimal point was included in further statistical analysis.

### 2.10. Static Balance

Static balance refers to the ability to stand still on a stationary floor. The balance abilities were measured with the Sharpened Romberg (SR) Test with eyes open (EO) and eyes closed (EC). The test administrator initially showed the correct position. Then, the subject was instructed to choose the order of the front foot voluntarily and to stand in full tandem, heel-to-toe position, without shoes, and with arms crossed on the chest. The first two trials were performed with open eyes for familiarization reasons, and the subjects tried to maintain the position for 30 s. Time was stopped when the subject could not hold the correct position. The following two trials were made with eyes closed. The subject took the same position and voluntarily closed the eyes. The measurement started by the time the subject indicated readiness and closed the eyes. Safety during measurements was given by the test administrator. Again, time was stopped if the subject opened the eyes or could not maintain the correct position. The longest accomplished time with closed eyes was noted in seconds to one decimal point and further statistically analyzed. The interrater reliability (EO: ICC, r = 0.99, EC: ICC, r = 0.99) and test–retest reliability (EO: ICC, r = 0.91, EC, r = 0.77) are regarded to be high, and the test is considered valid for measuring static postural control in elderly [21].

### 2.11. Quality of Life

The quality of life was collected using the short version of the WHO’s quality of life (WHOQOL-BREF) questionnaire. The WHOQOL-BREF questionnaire is a reliable and valid instrument for identifying health-related QoL, as well as social, environmental, and subjective well-being issues [22]. WHOQOL-BREF consists of a background survey, and 26 quality-of-life items are included. The first two questions are mirroring general QOL and the overall satisfaction with health. Subsequent questions can be related to four different dimensions of QOL: Physical health (seven questions), psychological (six questions), social relationships (three questions), and the environmental dimension of QOL (eight questions). The questions of these dimensions were in a mixed order. The most appropriate option can be chosen as a response from a five-level Likert scale, where option 1, depending on the question, means a negative evaluation; for example, in the form of very dissatisfied, very poor, or not at all. Option 5, respectively, means a positive evaluation, such as very satisfied, completely, or always. The results of the survey were handled in accordance with the WHOQOL-BREF manual. In reporting, the scores were converted to a scale of 0–100, where higher scores indicate better QOL. The WHOQOL-BREF is a valid assessment tool for analyzing QOL when reflected by its four domains. It provides good-to-excellent reliability and validity [23]. In the study of Lucas-Carrasco, Laidlaw, and Power [24], the internal consistency for the WHOQOL-BREF total scale measured by Cronbach’s alpha was 0.90. The WHOQOL-BREF questionnaire concerning QOL was asked to be filled twice (pre and post).

### 2.12. Statistics

All primary (flexibility) and secondary (QOL, strength, and balance) outcomes are presented as means (M) with standard deviations (SD). Several repeated measure analyses of variance (rANOVA) were separately computed for each outcome measure. Thereby, INT vs. CON served as GROUP- variable and pre vs. post as the repeating factor TIME. Thereby, baseline values have been included as covariates. For pairwise comparison, multiple Tukey post hoc tests were calculated in case of significant interaction effects. Significance level was set at *p* < 0.05 *, *p* < 0.01 **, and partial eta squares (np^2^) were computed (trivial if 0 ≤ η_p_^2^ < 0.05; small if 0.05 ≤ η_p_^2^ < 0.24; a moderate effect if 0.25 ≤ η_p_^2^ < 0.64; and a strong effect if η_p_^2^ ≥ 0.64) for overall effect size estimation [25]. 

## 3. Results

### 3.1. Flexibility, Strength, and Balance

Data for pre- and post-testing are presented as means with standard deviations in Table 3. A statistically significant and moderate TIME x GROUP effect was observed for the primary outcome of the CSR test (Table 3). Subsequent post hoc pair-wise comparison revealed meaningful and significant differences from pre to post for the INT. In contrast, CON did not improve their CSR performance notably. Interestingly, shoulder flexibility measured with BS did not improve to a significant extent. Also, lateral flexibility of the spine remained nearly unchangeable and did not show significant changes after adjusting for baseline differences. 

Small but meaningful effects (η_p_^2^ = 0.18) with a trend to significance level (η_p_^2^ = 0.06) favoring INT were also observed for lower-extremity strength testing employing the 5STS test (Table 3). Subsequent post hoc testing revealed significantly improved test times at post- compared to pre-testing for INT (*p* = 0.003). Despite large baseline differences, rANOVA analyses also showed small but meaningful interaction effects for static balance with closed eyes. Follow-up post hoc testing revealed significant improvements in favor of INT (Table 3). 

### 3.2. Quality of Life (QoL)

With mainly small effect sizes, overall QoL did not show notable differences between group effects (Table 4). However, the sub-domain referring to the environment revealed a small but significant effect in favor of INT. Also, subsequent post hoc testing revealed significant differences between pre- and post-testing for INT, whilst CON remained unchanged. 

## 4. Discussion

The aim of this two-armed randomized controlled trial was to gain new insights into the potential effects of sauna yoga on relevant physical health outcomes (flexibility, strength, and balance) in healthy and relatively active older adults. To the best of our knowledge, no previous studies addressed this issue in a controlled study with parallel study arms, assessing both physical dimensions and quality of life. It was hypothesized that sauna yoga can beneficially affect spinal, shoulder, and hamstring flexibility, whereas only little effects on lower-extremity strength and static balance performance were expected. The results of this study indicated that, after only eight session over eight weeks, flexibility using the CSR test notably improved in favor of the INT group, whereas shoulder flexibility and lateral spinal flexibility were not relevantly affected. Interestingly, small but meaningful changes in favor of the INT group with slightly significant results were also found for lower-extremity strength and static balance with closed eyes. Thus, our previously postulated hypothesis was only partly confirmed. 

In the light of previous research on yoga training, significant improvement in flexibility could have been expected. However, only few studies have been undertaken in the elderly population. In comparison to the study of Gonçalves, Vale, Barata, Varejão, and Dantas [26], which showed a significantly improved range of motions (ROM) in the shoulder girdle and in the spine, our study applied a comparatively shorter intervention period, lower weekly training intensity, and a moderately warm environment of 50 °C.

The effects of chair yoga in ambient temperature without using a sauna environment was studied in the past, but mostly in relation to psychological dimensions, like fear of falling or quality of life. For example, Furtado et al. [27] were not able to show any improvements in physical fitness outcomes after 14 weeks of chair yoga in a group of institutionalized older women. Furthermore, Park, McCaffrey, Dunn, and Goodman [28] were more focused on the assessment of clinical symptoms perception of osteoarthritis. 

Based on the findings of our study, performing yoga poses in a seated position in a warm environment can superiorly improve flexibility in healthy community-dwelling older adults. The current study is thus partly in line with earlier studies that revealed effects of yoga on strength and balance in the elderly [29,30]. However, compared to many yoga studies, the weekly intensity of the present intervention was comparatively low. In other yoga studies, yoga classes were performed two to three times weekly [30,31,32]. Additionally, the session duration was longer than in sauna yoga, and it was also longer than in the hot yoga interventions [33,34]. However, the participants were younger (46 ± 12; 53 ± 2 years) than the ones in our intervention group (69 ± 5 years). Furthermore, it should be taken into consideration that the room temperature in sauna yoga is approximately 10 °C higher than in hot yoga and 30 °C higher than in normal yoga. With more frequent sauna yoga sessions, the improvements could have been more pronounced from a dose-response perspective. On the other hand, normal sauna visits are also performed once to twice weekly, for approximately 15 min [35].

Results of a six-month yoga intervention study in the elderly indicated beneficial effects on physical health, psychological health, social relationships, and environmental domains of QoL [36]. Gonçalves et al. [26] showed significant changes in overall QoL and in the dimensions of physical health and environment. However, in the present study, significant effects were detected only in the environmental domain of QoL in favor of the INT group, and the effects were smaller than in the study of Hariprasad et al. [36] or Gonçalves et al. [26]. The participants of this study were moderately or highly active, which may affect detectability in QoL, as the QoL is associated with physical independency and mobility.

Dewhurst and Bampouras [37] recommended that when measuring flexibility with CSR or BS, both sides should be measured separately, so that the side differences can be detected. In this study, the participants could choose themselves which side they wanted to have measured. However, the side was noted, and the same side was measured in the post measurements. CSR and BS are highly reliable when repeated and executed within a short period [37]. As recommended, both tests were performed twice, without familiarization. In future studies, both sides should be included in the measurement, to produce more detailed information on the side differences and flexibility imbalance.

Standing balance testing is reported to be rather easily applicable for community-dwelling adults [21] and a ceiling-effect in standing balance with closed eyes is not likely. In this regard, testing static balance with EC is noted to be very sensitive to the influence of age [38], and the time limit of 10 s with EC is adopted in some geriatric studies [39,40]. It was repeatedly emphasized that, in the future, more specific tests need to be chosen for assessing static balance, e.g., center of pressure path length at force plate. The current study did not measure the changes in trunk strength, even though it is associated with postural control [41]. Additionally, many poses aimed to improve trunk strength. This test parameter should be considered in future studies concerning sauna yoga and strength. 

## 5. Conclusions

Although the sample size of this study refers to a pilot character, we were able to detect significant and meaningful change in the primary endpoint. Thus, the sample size can be regarded as sufficient. Initially, we intended to examine the feasibility of using yoga in a warm sauna environment to affect physical health outcomes in a group of healthy, active seniors. When considering the present data, general neuromuscular transfer effects to flexibility, strength, and balance can be expected. The strongest improvements have been found in flexibility, as this part was the main focus of attraction throughout the training sessions. Against this background, the authors recommend further studies on sauna yoga in order to gain more information on training interventions that can improve overall long-term mobility, strength, and balance in healthy elderly subjects. The study also suggests that the effects of sauna yoga should be studied with a greater number of participants and a longer intervention period, with increased weekly intensity. 

During the intervention, none of the participants complained about inconvenience or negative effects regarding sauna yoga. On the contrary, only positive feedback was given, and participants enjoyed and looked for further sessions. Therefore, it is recommended that sauna yoga under supervision is a suitable activity for healthy older adults and can be incorporated into an activity program in spas, fitness centers, and senior activity centers. Additionally, the results of current this research may lead to a new perspective on thermal therapy as treatment for diseases that decrease ROM (e.g., arthrosis and rheumatism).

## Figures and Tables

**Table 1:** Participants’ data for intervention (INT) and control (CON) groups. Data provided as means ± standard deviations (SD).

	INT	CON
Gender (f/m)	10/1	9/1
Age (years)	68.7 ± 5.9	69.3 ± 4.9
Height (cm)	166.6 ± 7.3	169.0 ± 5.1
Weight (kg)	66.9 ± 9.4	67.6 ± 9.0
Body Mass Index (kg/m^2^)	24.3 ± 2.6	23.7 ± 2.7
Physical Activity (MET/week)	199.0 ± 80.9	182.0 ± 84.0

**Table 2:** Performed exercises during the intervention. Data provided as sets and repetitions or duration.

Exercise	Week 1	Week 2	Week 3	Week 4	Week 5	Week 6	Week 7	Week 8
0	1 min	1 min	1 min	1 min	1 min	1 min	1 min	1 min
1	2 × 3	2 × 3	2 × 4	2 × 4	2 × 4	2 × 4	2 × 4	2 × 4
2	2 × 2	2 × 2	2 × 4	2 × 4	2 × 4	2 × 4	2 × 4	2 × 4
3	1 × 30 s	2 × 30 s	2 × 30 s	3 × 30 s	3 × 30 s	3 × 30 s	4 × 30 s	4 × 30 s
4	2 × 30 s	2 × 30 s	2 × 30 s	2 × 30 s	2 × 30 s	2 × 30 s	2 × 30 s	2 × 30 s
5a	1 × 4	2 × 4	1 × 4	1 × 4	1 × 8	1 × 8	1 × 8	1 × 8
5b	-	-	1 × 30 s	2 × 30 s	3 × 30 s	3 × 30 s	3 × 30 s	3 × 30 s
6	1 × 4	1 × 4	1 × 4	1 × 4	1 × 4	1 × 4	1 × 4	1 × 4
0	1 min	1 min	1 min	1 min	1 min	1 min	1 min	1 min

Exercises: 0-relaxation and meditation, 1-shoulder and neck flexibility, 2-spine flexibility, 3-leg strength, 4-spine rotation, 5-core 1 and 2, and 6-hip flexibility.

**Table 3:** Data for pre- and post-testing for the sauna yoga group (INT) and the control group (CON). The rANOVA interaction effects were calculated with baseline values included as covariates, and effect sizes are given as partial eta squared (η_p_^2^).

	INT		CON		GROUP × TIME Interaction	
Test	Pre	Post	Pre	Post	*p*	η_p_^2^
**CSR**	7.86 ± 7.86	16.70 ± 9.08 **	3.65 ± 15.30	7.80 ± 13.60	0.028 *	0.241
BS	−5.34 ± 10.70	−2.05 ± 5.72	−4.15 ± 5.69	−2.90 ± 13.10	0.129	0.130
LF_R	44.90 ± 4.54	44.60 ± 4.92	46.50 ± 6.53	45.0 ± 6.46	0.379	0.043
FL_L	46.50 ± 4.49	44.90 ± 4.57	46.10 ± 5.20	45.0 ± 4.62	0.713	0.008
5STS	7.26 ± 1.80	5.90 ± 1.20 **	6.73 ± 1.93	6.73 ± 1.93	0.061	0.181
SR_EC	14.60 ± 12.40	22.0 ± 11.0 *	5.54 ± 12.40	7.80 ± 6.54	0.056	0.189

Chair sit and reach (CSR); back scratch (BS); lateral flexion right/left (LF_R/LF_L); 5 times sit-to-stand (5STS); Sharpened Romberg eyes open/eyes closed (SR_EO/SR_EC); significance level was set at *p* < 0.05 *, *p* < 0.01 **.

**Table 4:** Reported QoL data of INT and CON groups. Data (pre, post) are provided as means with standard deviations (SD). The *p*-values are calculated with the analyses of variance (ANOVA) and effect sizes are given as partial eta squared (ηp2).

	INT		CON		GROUP × TIME Interaction	
QoL	Pre	Post	Pre	Post	*p*	η_p_^2^
All	7.55 ± 1.58	8.36 ± 1.12	8.60 ± 1.07	8.80 ± 1.03	0.176	0.099
PH	76.30 ± 18.40	82.00 ± 11.30	83.50 ± 10.03	84.60 ± 9.50	0.651	0.012
PSY	69.90 ± 11.00	75.70 ± 9.78	70.60 ± 14.40	79.30 ± 10.30	0.359	0.047
SR	63.30 ± 13.50	72.80 ± 9.02	70.60 ± 12.30	73.10 ± 11.50	0.279	0.065
ENV	80.30 ± 12.00	86.00 ± 7.07 *	85.20 ± 6.91	85.10 ± 8.03	0.034 *	0.227

Quality of life (QoL); overall satisfaction (All); physical health (PH); psychological dimension of QOL (PSYCH); social relationships (SR); environmental dimension of QoL (ENV); significance level was set at *p* < 0.05 *.
